# Mental health and work: a European perspective

**DOI:** 10.1017/S2045796024000246

**Published:** 2024-04-05

**Authors:** Angelo Fioritti, Hlynur Jònasson, Lars de Winter, Chantal Van Audenhove, Jaap van Weeghel

**Affiliations:** 1 IPSILON Association, Bologna, Italy; 2 Landspitali, Psychiatric Hospital, Reykjavik, Iceland; 3 Phrenos Center of Expertise, Utrecht, The Netherlands; 4 Center for Care Research and Consultancy, Department of Public Health and Primary Care, Leuven University, Leuven, Belgium; 5 Tranzo Scientific Center for Care and Welfare, Tilburg School of Social and Behavioural Sciences, Tilburg University, Tilburg, The Netherlands; 6 MIND Netherlands, The National Organization for Mental Health Information, Support and Advocacy, Amersfoort, The Netherlands

**Keywords:** health economics, mental health, occupational psychiatry, social and political issues, social inclusion

## Abstract

Among the many social determinants of health and mental health, employment and work are getting momentum in the European political agenda. On 30–31 January 2024, a ‘High-level Conference on Mental Health and Work’ was held in Brussels on the initiative of the rotating Belgian Presidency of the European Union. It addressed the issue developing two different perspectives: (1) preventing the onset of poor mental health conditions or of physical and mental disorders linked to working conditions (primary prevention); (2) create an inclusive labour market that welcomes and supports all disadvantaged categories who are at high risk of exclusion (secondary and tertiary prevention). In the latter perspective, the Authors were involved in a session focused on ‘returning to work’ for people with mental disorders and other psychosocial disadvantages, with particular reference to Individual Placement and Support as a priority intervention already implemented in various European nations. The themes of the Brussels Conference will be further developed during the next European Union legislature, with the aim of approving in 4–5 years a binding directive for member states on Mental Health and Work, as it is considered a crucial issue for economic growth, social cohesion and overall stability of the European way of life.

## Introduction

Our post-pandemic era seems to have brought two themes back to the forefront of political discussion and public opinion, among other things: the meaning of work and the value of mental health. It is therefore comprehensible that a third theme that combines them is acquiring great importance, namely that concerning the relationship between mental health and work.

On 30–31 January 2024, the ‘High level Conference on Mental Health and Work’ was held in Brussels on the initiative of the rotating Presidency of the European Union, which saw the participation of around 200 delegates and over 30 politicians including ministers, European Commissioners, Members of the European Parliament and national deputies. The Conference was created to follow up on the action undertaken in the previous semester by the Spanish Presidency (with particular commitment from Labour Minister Yolanda Diaz) who had given priority to the topic, managing among other things to produce a declaration from the Council of Europe on 9 October 2023 on ‘Mental health and precarious work’ (Council of the European Union, 2023). The Belgian Presidency intended to relaunch the topic with the aim of arriving at a binding European directive within the necessary institutional times (a few years) that supports the efforts of the member states in two directions: (1) preventing the onset of poor mental health conditions or of mental disorders linked to working conditions (primary prevention); (2) create an inclusive labour market that welcomes and supports all disadvantaged categories who are at high risk of exclusion (secondary and tertiary prevention). In the latter perspective, the Authors were involved in a session focused on ‘returning to work’ for people with mental disorders and other psychosocial disadvantages, with particular reference to Individual Placement and Support (IPS) as a priority intervention already implemented in various European nations (Jònasson *et al.*, 2022) and favourable conditions for its further development. With this editorial the authors intend to outline the conceptual framework within which the technical debate on the topic of mental health and work is developing, with its implications in terms of labour, employment and mental health policies.

## Why is this topic being addressed now?

The political level at which the Brussels Conference was placed suggests that it has acquired a notable priority in the Union agenda. The works were opened by two European commissioners (Margaritis Schinas, vice-president of the European Commission and Commissioner to the European Way of Life, and Stella Kyriakides, European Commissioner for Health and Food Safety), by the Deputy Prime Minister of Belgium (Petra De Sutter, minister for public administration). During the proceedings also spoke the German Labor Minister (Lilian Tschan), the Spanish Health Minister (Javier Padilla) and Members of European Parliament (MEPs) from various countries.

From their interventions it was clear that the political theme of the meaning and quality of work in contemporary and future Europe is of great relevance with regard to economic growth, social cohesion and the overall stability of the European model of life. The challenges posed by the pandemic, digitalisation, globalisation of markets, growing precariousness of work and reduction in the negotiating power of trade unions have been recalled several times. These changes are producing both worrying health and economic consequences, such as rising rates of sick leaves, absenteeism, presenteeism, increased direct medical and indirect social costs and higher rates of population in need of assistance. All this happens in the scenario of economic competition with areas of the world such as Asia and Africa which remain far from the quality standards at work that Europe pursues. In essence, there is a well-founded fear that the European dream will be shattered when there are large sections of the population excluded from work or employed in precarious, alienating, underpaid jobs marked by mere exploitation.

The Brussels Conference seems to be a downstream response to the problem, hopefully combined with upstream responses regarding the economic cycle, labour policies, trade union protections, international trade policies, social and health policies. However, it represents the awareness of the risks that masses of excluded or alienated people can pose for our society, with imaginable political consequences and social conflict. It is worth mentioning that the works took place in a building adjacent to the Council of Europe and the European Commission while the large farmers’ demonstration was being prepared with the tractors already lined up in the near streets. A tangible demonstration of what the value of social cohesion means and the political consequences of its loss.

It should also be noted that in 2023 the European Commission addressed a specific communication to the European Parliament regarding an overall strategy for mental health based on three pillars (prevention, services and inclusion) which sees mental health in the workplace as one of the fundamental actions. This declaration partly takes up the contents of the work carried out within the Joint Action on Mental Health and Wellbeing (2012–2016) which had produced three specific documents related to the current issue: (1) Mental Health in the Workplaces, (2) Towards Community-based and Socially inclusive Mental Health Care and (3) Mental Health in all Policies. Furthermore, it must be noted that not only the European Union is setting mental health and work as a priority issue, but also the Organization for Economic Co-operation and Development (OECD, 2016) and the World Health Organization (WHO, 2022) are giving to the issue an unprecedented attention.

## Primary prevention: psychosocial risk, stress and burnout

This topic was addressed with the contribution of high-level experts from epidemiological, sociological, prevention, occupational health and work psychology backgrounds.

The European Institute of Trade Unions (Euro Trade Union Institute) commissioned the University of Quebec in Montreal to carry out a study on the burden of depression and cardiovascular diseases due to the so-called ‘psychosocial risk’ (i.e. unwell conditions linked to work). The results are published in the main international scientific journals of preventive medicine and public health and are of great impact (Niedhammer and Chastang, 2021; Sultan-Taıeb *et al.*, 2022). The psychosocial risk linked to exposure at work is thought to be responsible for approximately 8% of the coronary heart diseases that occurred in Europe in 2015 and for approximately 28% of all cases of depressive illness. There are notable differences between member states, with a gradient that for coronary heart disease sees the highest rates in Eastern countries with a progressive decrease moving westward. In relation to direct costs (medical treatment and drugs) and indirect costs (absenteeism, presenteeism, years of life lost, etc.), an impact of 11–15 billion euros is estimated for cardiovascular diseases and 45–90 billion euros for depression .

The European Agency for Occupational Health and Safety has provided data on the health impact of psychosocial risk related to exposure at work. It is estimated that 10% of all Disability Adjusted Life Years (DALYs) in the European Union are linked to problems at work, mainly related to stressful working times (46%), poor communication (26%), poor work autonomy, physical or verbal abuse and bullying. There are profound country-specific differences and challenges between EU countries that call for specific national action plans and legislations. Many companies have started to have their own action plans to reduce psychosocial risk in the workplace, also encouraged by national legislation which is in some cases binding.

On the topic of psychosocial risk, three parallel sessions were held, dedicated to the contributions of occupational psychology, of labour law and of occupational medicine respectively. The political debate saw dialogue (the most used term was ‘social dialogue’, i.e. trade union relations) between representatives of workers (European Trade Union Confederation), of large companies (Business Europe), of companies that supply public services (Services of General Interest) and small and medium-sized enterprises (UNIZO – Small Medium-size Enterprises United). The debate concerned the role of national legislation (especially on working hours and shifts), of labour inspectors, on rules for working from home, on digitalisation and on worker control systems through production algorithms. The constant calls to keep the ‘social dialogue’ open have left the impression that there is still a lot of work to be done.

## Secondary and tertiary prevention: the inclusive labour market (start-stay-return to work)

Compared to the previous theme, the scientific contribution of academic study centres and international organisations was significantly lower. At a European level, there are no real data on the employment rates of populations at high risk of exclusion from the labour market such as people with mental disorders, addictions, disabilities and migrants. The contributions were mostly of a political and legislative nature, with references to the international conventions, to the European standards and to the three pillars of the European approach: (1) quality jobs, (2) adaptations at work (flexible hours, job redesign, reasonable accommodations, etc.) and (3) integration of assistance and work.

The parallel sessions concerned: (1) start to work (place reservation policies, activation policies, training); (2) stay to work (occupational risk, attention to self-employment, psychological support, integration with healthcare) and (3) return to work with a particular emphasis on European experiences with IPS introduced and discussed by the Authors.

IPS is a model developed in the ‘90s in the USA in order to support people with severe mental disorders to enter the competitive labour market (Becker and Drake, 2003). It entails a ‘place and support’ approach, rather than a traditional ‘train and place’ one, and the integration of employment support and mental healthcare. Its excellent results in the USA have been confirmed by several controlled studies in the USA (Bond *et al.*, 2020) and in Europe (Burns *et al.*, 2007; Fioritti *et al.*, 2014), despite relevant differences in welfare systems and labour market regulations. In this last session there were four contributions (Hlynur Jònasson, Lars De Winter, Saskia Decuman and Angelo Fioritti) commenting on the main report by Jaap van Weeghel. A lively debate followed, which was also resumed in the plenary session, which highlighted the lack of penetration of IPS in many European countries and the opportunities for IPS to become a reference model in mental health and labour policies.

## Conclusions

What remains of this European stage in terms of mental health and work?

It was quite clear that the push to deal with the issue comes from problems within the world of work, stimulated as it is by internal and international competition, technical and technological complexity, digitalisation, specialisation, difficulty in reconciling family and work, precariousness. The urgency of these problems is also recognised by mental healthcare services, that are confronted with the extensive negative consequences of mentally unhealthy work conditions.

What Europe would like to do is to create a framework in which businesses and work organisations can collaborate (‘social dialogue’) in close collaboration with Mental Health Care services to reduce malaise (‘psychosocial risk’) linked to the ‘status syndrome’, the psychological experience of inequality – how much control people have over their lives and their opportunities for full social participation that has a profound effect on their physical and mental health (Marmot, 2006). If the Union is able to support social cohesion this way, it may prevent large masses of the population from entering into a spiral of alienation and becoming easy prey to extremism and populism.

It is positive that this orientation has also linked the theme of the involvement of populations at greater risk of exclusion, such as people with mental illness, addictions and disabilities. The investment in terms of research and development of solutions in this second field seems much smaller than the first. Even more so, the European experiences on IPS can present themselves as an effective and sustainable model, susceptible to rapid implementation and experiments with appropriate adaptations for populations equally at risk. The recently established European IPS Learning Community Network can represent an important basis for further development of IPS in Europe, possibly with the support of European institutions.

Announced next step is the development of a ‘peer-review’, i.e. a document that will allow to start a political debate within the next Commission and the next EU Parliament. And maybe, at the end of this debate to draw and approve a European Directive on Mental Health and Work. Time foreseen for the whole process is 4–5 years.
